# Constitutive activation of integrin αvβ3 contributes to anoikis resistance of ovarian cancer cells

**DOI:** 10.1002/1878-0261.12845

**Published:** 2020-12-01

**Authors:** Romana Dolinschek, Julia Hingerl, Anke Benge, Christian Zafiu, Elisabeth Schüren, Eva‐Kathrin Ehmoser, Daniela Lössner, Ute Reuning

**Affiliations:** ^1^ Department for Obstetrics & Gynecology Clinical Research Unit Technische Universität München Germany; ^2^ Department of Water, Atmosphere, and Environment University for Natural Resources and Applied Life Sciences (BOKU) Vienna Austria; ^3^ Department for Nanobiotechnology Institute for Synthetic Bioarchitectures University for Natural Resources and Applied Life Sciences (BOKU) Vienna Austria; ^4^ Faculties of Engineering and Medicine Nursing & Health Sciences Monash University Melbourne Vic. Australia

**Keywords:** apoptosis/anoikis resistance, constitutive integrin activation, integrin signaling, integrin transmembrane domain conformation, integrin αvβ3, ovarian cancer spheroid

## Abstract

Epithelial ovarian cancer involves the shedding of single tumor cells or spheroids from the primary tumor into ascites, followed by their survival, and transit to the sites of metastatic colonization within the peritoneal cavity. During their flotation, anchorage‐dependent epithelial‐type tumor cells gain anoikis resistance, implicating integrins, including αvß3. In this study, we explored anoikis escape, cisplatin resistance, and prosurvival signaling as a function of the αvß3 transmembrane conformational activation state in cells suspended in ascites. A high‐affinity and constitutively signaling‐competent αvß3 variant, which harbored unclasped transmembrane domains, was found to confer delayed anoikis onset, enhanced cisplatin resistance, and reduced cell proliferation in ascites or 3D‐hydrogels, involving p27^kip^ upregulation. Moreover, it promoted EGF‐R expression and activation, prosurvival signaling, implicating FAK, src, and PKB/Akt. This led to the induction of the anti‐apoptotic factors Bcl‐2 and survivin suppressing caspase activation, compared to a signaling‐incapable αvß3 variant displaying firmly associated transmembrane domains. Dissecting the mechanistic players for αvß3‐dependent survival and peritoneal metastasis of ascitic ovarian cancer spheroids is of paramount importance to target their anchorage independence by reversing anoikis resistance and blocking αvß3‐triggered prosurvival signaling.

## Graphical abstract

EOC cells are shed from the primary tumor and survive the transit in ascites to colonize at metastatic sites. In EOC cell transfectants, a constitutively active αvß3 with unclasped TMD conferred anoikis resistance via prosurvival signaling implicating the activation of the EGF‐R, FAK, src, PKB/Akt, and Erk, compared with rapid onset of apoptosis by a signaling‐incompetent αvß3 with associated TMD.

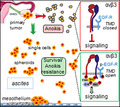

**Abbreviations**
CLSM: confocal laser scanning microscopy
ECM: extracellular matrix
EGF‐R: epidermal growth factor receptor
EOC: epithelial ovarian cancer
FAK: focal adhesion kinase
FIGO: Fédération Internationale de Gynécologie et d'Obstétrique
GAPDH: glyceraldehyde 3‐phosphate dehydrogenase)
GpA: glycophorin A
IMD: integrin‐mediated death
MAPK: mitogen‐activated protein kinases
PI: propidium iodide
RGD: Arg‐Gly‐Asp
TMD: transmembrane domain

## 1 Introduction

Epithelial ovarian cancer (EOC) has a high mortality rate that is mainly due to late diagnosis when peritoneal metastasis has already occurred. Early steps of EOC progression involve the disruption of the ovarian tumor capsules, followed by the shedding and dissemination of EOC cells in ascites, representing a frequent clinical complication for EOC treatment [1, 2].

Most nontransformed epithelial‐type cells attach to the extracellular matrix (ECM) via binding by cell adhesion receptors of the integrin superfamily. Integrins recognize in ECM proteins, for example, the tripeptide motif Arg‐Gly‐Asp (RGD). This provokes bidirectional signaling (*outside‐in* and *inside‐out)* regulating cell survival, differentiation, proliferation, and motility. Thus, abrogation of ECM interactions by unligated integrins has a decisive impact on cell survival, triggering a special form of apoptosis, the so‐called anoikis, via the integrin‐mediated death (IMD) [3, 4, 5]. However, malignant cells, including EOC cells, are capable of resisting anoikis by acquiring anchorage independence during their spread and eventual anchoring to the mesothelial ECM to allow their metastatic colonization [1, 6, 7, 8, 9]. During these processes, floating cancer cells modulate their cell–cell adhesive contacts forming multicellular spheroids and change their expression of protein kinases, phosphatases, integrin‐related signaling molecules, and anti‐/proapoptotic factors, for example, of the Bcl‐2 protein family [10, 11, 12, 13, 14]. Also, cysteine proteases of the caspase family are important players in cancer cell survival and tumor progression. The intrinsic and extrinsic apoptotic cascades converge on the level of the executioner caspase‐3, which upon its activation triggers a proteolytic cascade, leading to distinct cell morphological and biochemical changes upon degradation of proteins and DNA [15].

Since certain integrins play a major role in cell survival and protection from apoptosis [16, 17, 18, 19, 20], tumor cell strategies to escape anoikis are thought to implicate the constitutive activation of integrin‐associated signaling pathways, involving the focal adhesion kinase (FAK) [21], src kinases [22, 23], the PI3K/PKB/Akt kinases [24], and mitogen‐activated protein kinases (MAPK) [25, 26]. Hereby, αvβ3 takes over prominent functions in EOC progression and correlates with poor patient prognosis [27, 28, 29, 30, 31]. Its tumor biological role is also underlined by *in vitro* findings, documenting enhanced EOC cell adhesion, migration, and proliferation upon its overexpression and engagement by ECM ligands [30, 31, 32].

Here, we investigated in EOC cells suspended in ascites, differences in anoikis resistance as a function of the αvβ3 conformational activation state of its transmembrane domains (TMD) and the cellular signaling arising thereof. Molecular dynamics and experimental data have provided compelling evidence that during integrin activation, the conformation of the TMD and the cytoplasmic tails crucially affect integrin ligand binding affinity and signaling competence. In the resting inactive state, integrins exhibit bent extracellular domains, which bind ECM ligands with low affinity. This triggers further structural alterations within the α‐ and the β‐subunit involving the dissociation of the TMD and cytoplasmic regions. Upon binding of intracellular proteins, such as talin, to the ß‐cytoplasmic region, the extracellular domains erect and the TMD dissociate, resulting in a fully activated conformational integrin state with high ligand binding affinity and full signaling capability. Within this respect, computational studies revealed an integrin TMD conformation similar to that found in the well‐studied dimeric erythrocyte protein glycophorin A (GpA), which harbors the dimerization motif GxxxG. Indeed, such a GpA‐like sequence had been discovered in nearly all α‐ and β‐integrin subunits. A global search of TMD interactions within 16 different integrins demonstrated a GpA‐like conformation with lowest energy, suggesting integrin TMD heterodimerization via a GpA‐like mode [33, 34, 35, 36, 37].

As experimental cell model, we previously established EOC cell transfectants which express αvß3 in different GpA‐TMD conformational activation states, in the context of an otherwise unaltered αvβ3 molecule: (a) an αvß3 variant encompassing a firmly associated GpA‐TMD, conferring low affinity and signaling incompetence to αvß3 (TMD‐GpA) and (b) an αvβ3‐TMD variant harboring an unclasped GpA‐TMD provoked by the mutation of the GxxxG‐motif to GxxxI, known to abrogate TMD dimerization, resulting in a high‐affinity αvß3 receptor with constitutively active signaling capability (TMD‐GpA‐I) [32]. The enhancement of the understanding of the crucial role of integrins and their associated cellular players in anoikis resistance during cancer metastasis is of high clinical significance for the identification of new therapeutic strategies.

## 2 Materials and methods

### 2.1 Cell line and culture

Origin, culture, and authenticity of human OV‐MZ‐6 ovarian cancer cells had previously been described. Cells were either grown under adherent conditions as described earlier [30, 32] or in suspension under floating conditions in ascites or cell culture medium (DMEM) where they form spheroid‐like cell aggregates.

### 2.2 Stable cell transfections

Stable OV‐MZ‐6 cell transfectants overexpressing αvβ3 wild‐type (TMD‐αvβ3) or its two GpA‐TMD chimers TMD‐GpA and TMD‐GpA‐I were generated as described earlier [32]. In initial experiments, vector controls almost lacking αvβ3 were included in the experiments leading to the same results as outlined in the previous detailed characterization of our EOC αvβ3 cell transfectant model within the preceding project [32], disclosing no significant αvβ3‐relevant effects. Based on these observations and because specific differences in αvβ3 biology depending on its different TMD conformations were in the focus of this work, we defined TMD‐αvβ3 as the appropriate control to judge functional consequences of altered TMD conformations for the αvβ3 receptor [32].

### 2.3 Annexin‐V‐Fluos/PI apoptosis assays

Cells were grown under floating conditions in ascites or DMEM for 48 h. Malignant ascites was collected from 5 different patients afflicted with high‐grade serous ovarian cancer (FIGO IIIb/c/IV) under sterile conditions, centrifuged at 300 ***g*** for 10 min and cell‐free supernatants stored at −80 °C. The collection and the analysis of ascites were approved by the ethics committee of the Medical Faculty of the Technical University in Munich. Donors provided written consent for the use of their sample for research purposes in accordance with the Declaration of Helsinki. For the detection and quantification of vital, apoptotic, and necrotic cells, staining with Annexin‐V‐Fluos and propidium iodide (PI; Roche Diagnostics GmbH, Mannheim, Germany) was performed according to the manufacturers' recommendations. Furthermore, cells were grown suspended in ascites or DMEM for 30 h and treated with cisplatin (2.5 µg·mL^−1^, provided by the central pharmacy of the Klinikum rechts der Isar, Technische Universität München) for another 18 h. These stained cell samples were also inspected by confocal laser scanning microscopy (CLSM) using the Axio Observer. Z1 + LSM700 microscope (Zeiss, Oberkochen, Germany) applying the zen‐software.

### 2.4 Measurements of caspase activation

Cleaved‐activated caspase‐3 was detected in cell transfectants grown for 48 h in suspension by western blot analysis using a specific mAb (Cell Signaling Technology, Frankfurt, Germany), according to the manufacturer`s protocol and by immunofluorescent staining upon use of an Alexa 488‐labeled secondary antibody (Thermo Fisher Scientific, Carlsbad, CA, USA). In addition, caspase activation was detected indirectly, by measuring its cleavage products of cytokeratin‐18 utilizing the M30 CytoDeath FACS kit (Roche Diagnostics GmbH) and by CLSM. Moreover, activated caspase was detected by binding of an irreversible fluorescence‐labeled pan‐caspase inhibitor applying the CaspACE™ FITC‐VAD‐FMK in Situ Marker kit (Promega, Madison, WI, USA).

### 2.5 Cell adhesion assay

The adhesive capacity of OV‐MZ‐6 cells was assessed as previously described [30, 32].

### 2.6 Cell proliferation assays

#### 2.6.1 Cells in suspension

Cells were cultivated in ascites for 3 and 48 h, respectively, and cell numbers determined by counting under trypan blue exclusion, following a short treatment with trypsin on ice to disassemble floating cell clusters for accurate cell counting.

#### 2.6.2 Cell proliferation in 3D‐hydrogels

Cells were encapsulated in PEG‐based proteolytic degradable hydrogels (QGel, Lausanne, Switzerland) in 24‐well plates at a density of 3.5 × 10^5^ cells·mL^−1^ in the presence or absence of RGD peptides for 1, 6, 10, and 14 days, respectively, with regular medium replacements. Cell morphology was assessed by bright‐field microscopy and CLSM. For immunofluorescence imaging, cells were fixed in 4% (w/v) paraformaldehyde (PFA) in phosphate‐buffered saline (PBS) followed by 0.2% (v/v) Triton X‐100 in PBS. F‐actin filaments were stained with rhodamine 415‐conjugated phalloidin (0.3 U·mL^−1^) and nuclei with DAPI (2.5 μg·mL^−1^, Thermo Fisher Scientific) in PBS containing 1% (w/v) bovine serum albumin (BSA). Fluorescence signal intensity was visualized and photographed using a SP5 confocal microscope (Leica, Heidelberg, Germany). Z‐stacks were acquired with a constant thickness of 2 μm in order to generate a cross‐sectional profile of 100–150 equidistant XY‐scans. Cell metabolic activity and proliferation were determined using Alamar Blue and CyQuant assays as previously reported [38]. Briefly, samples were incubated with 4% (w/v) Alamar Blue reagent in phenol red‐free DMEM for 6 h at 37 °C/5% (v/v) CO_2_ and fluorescent signals (excitation 544 nm, emission 590 nm) detected using a plate reader. Fluorescence signal intensity was normalized to day 1. For CyQuant analysis, samples were frozen at −80 °C prior to digestion with proteinase K (0.5 mg·mL^−1^) in phosphate‐buffered EDTA overnight at −65 °C. Samples were then treated with RNase A (1.4 U·mL^−1^) in lysis buffer for 1 h at room temperature, a GR‐dye solution added, and fluorescence signal intensity (excitation 485 nm, emission 520 nm) detected using a plate reader. A DNA standard curve (0–2 μg·mL^−1^) was used to calculate the fold change of the DNA content per sample and normalized to day 1. Experiments were performed in triplicate hydrogel samples in three biological replicates (*P < 0.05; **P < 0.01).

### 2.7 Western blot analyses

#### 2.7.1 Detection of the (p‐) epidermal growth factor receptor (EGF‐R), (p‐)FAK, (p‐)p44/42^(erk‐1/2)^, (p‐)PKB/Akt, or (p‐)Src

Cell transfectants were grown for 48 h under adhesion or suspension and cellular proteins extracted and processed for western blot analysis as previously described using the ECL^TM^ chemiluminescent substrate (Pierce, Rockford, IL, USA) [32]. Detection of Bcl‐2, survivin, or p27^kip1^ was performed using monoclonal antibodies (mAb; Becton Dickinson Biosciences, Franklin Lakes, NJ, USA) which were diluted in Tris‐buffered saline, 0.1% (v/v) Tween‐20, 3–5% (w/v) BSA, and incubated with blot membranes overnight at 4 °C. Differences in protein loading and blotting efficiency were normalized by reprobing the membranes with a mAb directed to glyceraldehyde‐3‐phosphate dehydrogenase (GAPDH). Band signal intensities were evaluated by use of the Bio‐Rad Imager Gel Doc™ XR + and ChemiDoc™ XRS + Systems with the software Image Lab™ and normalized to those recorded for GAPDH.

### 2.8 Statistical data analysis

For the data corresponding to the proliferation assays in 3D‐hydrogels, independent *t*‐tests were performed with *P*‐value less than 0.05 indicating statistically significant differences (*P < 0.05; **P < 0.01). All other data were statistically analyzed by Sigma Plot 14 (Systat Inc., Erkrath, Germany) using one‐way or two‐way ANOVA with a *post hoc* pairwise significance test. The datasets were tested for normality (Shapiro–Wilk; condition *P* ≥ 0.050) and for equal variance (Brown–Forsythe; condition *P* ≥ 0.050). In case of passed tests, the ANOVA was performed parametrical with *post hoc* pairwise Bonferroni *t*‐test. Otherwise the analysis was performed with a Kruskal–Wallis ANOVA on ranks with *post hoc* pairwise Tukey *t*‐test. Significant differences are indicated by * or ^#^ for *P* < 0.050, ** or ^##^ for *P* < 0.010, and *** or ^###^ for *P* < 0.001 in graphical artwork.

## 3 Results

**Fig. 1:**
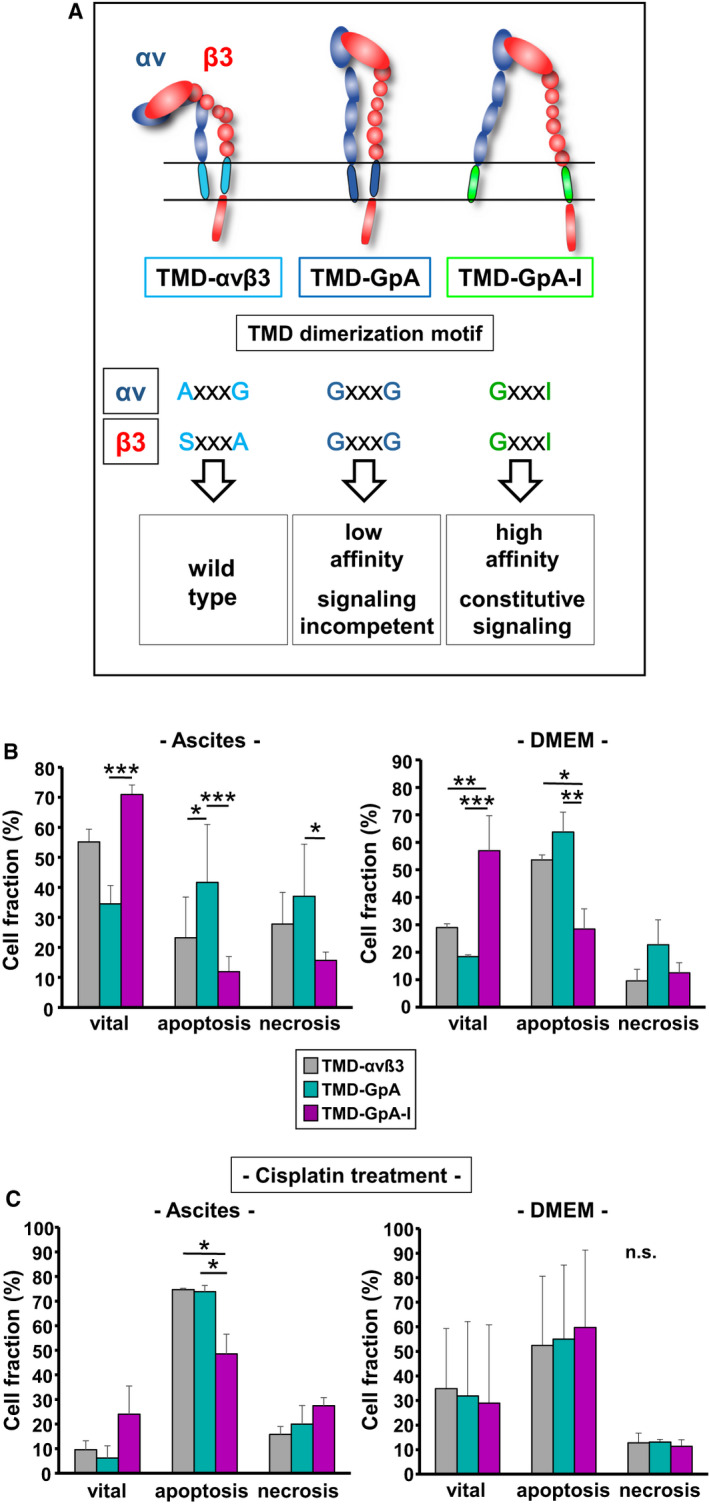
Detection of early apoptotic events in floating human EOC cells as a function of the αvβ3 TMD conformational activation state. (A) EOC αvβ3 cell transfectant model. Integrin αvβ3 TMD variants were generated by exchanging the complete TMDs of the αv‐ and the β3‐subunit by the strongly dimerizing TMD of GpA (TMD‐GpA), resulting in a low affinity αvβ3 receptor lacking signaling capability. In addition, we point‐mutated the dimerization motif within GpA, GxxxG, to GxxxI, known to abrogate TMD association, conferring high‐affinity and constitutively active signaling competence (TMD‐GpA‐I; published in ref. [32]). (B) Human EOC cell transfectants were cultured in suspension for 48 h in ascites or DMEM and subjected to Annexin‐Fluos V/PI apoptosis assays in order to discriminate vital, apoptotic, and necrotic cell fractions. (C) Cell transfectants suspended for 30 h either in ascites or in DMEM were treated for another 18 h by cisplatin. Data are given for each cell fraction in % as mean values ± SD from 3 independent experiments. Significance was tested by two‐way ANOVA with *post hoc* Bonferroni *t*‐test. Significance is indicated by *P < 0.050, **P < 0.010, and ***P < 0.001 (n. s., not significant).

In detached EOC cells, we set out to explore anoikis resistance, integrin signaling, and anti‐apoptotic factors as a function of the TMD conformational activation state of the tumor biologically relevant integrin αvβ3. We used our previously established and characterized *in vitro* EOC αvβ3 cell transfectant model [32]: (a) TMD‐αvß3: wild‐type αvß3; (b) TMD‐GpA: αvß3 displaying associated GpA‐TMD with low affinity and lack of signaling capacity; and (c) TMD‐GpA‐I: αvß3 with unclasped GpA‐TMD due to a point mutation in the dimerization motif GxxxG exhibiting high‐affinity and constitutive signaling competence (Fig. 1A).

### 3.1 Detection of early apoptotic events as a function of the αvβ3‐TMD conformational activation state

EOC cells grown for 48 h either suspended in ascites or in DMEM (Fig. 1B) were harvested and vital, apoptotic, and necrotic cell fractions discriminated by Annexin‐Fluos V/PI staining. Figure S1A depicts for all cell transfectants suspended in ascites a typical density plot quadrant analysis obtained by FACS. In ascites, for TMD‐GpA‐I expressers ~ 71% of cells were detected in the vital cell fraction, followed by ~ 55% for αvβ3 wild‐type (TMD‐αvβ3) transfectants, and ~ 34% for cells expressing TMD‐GpA. Apoptotic cell fractions were smallest for TMD‐GpA‐I (~ 12%), followed by TMD‐αvβ3 (~ 23%), and TMD‐GpA transfectants (~ 42%). The necrotic cell fraction was smallest for TMD‐GpA‐I (~ 16%), followed by TMD‐αvβ3 (~ 28%), and TMD‐GpA transfectants (~ 37%; Fig. 1B). When cells were allowed to float for 48 h in DMEM, ~ 58% of TMD‐GpA‐I transfectants were detected in the vital cell fraction, when compared to only ~ 18% vital TMD‐GpA transfectants. Cells harboring TMD‐αvβ3 disclosed an intermediate level of ~ 29% vital cells. TMD‐GpA‐I expressers exhibited a markedly lower percentage of apoptotic cells (~ 29%) when compared to TMD‐GpA transfectants (~ 63%) with a medium level of ~ 53% for TMD‐αvβ3 transfectants. Highest numbers of necrotic cells were detected for TMD‐GpA transfectants (~ 22%) with an almost comparable percentage of cells for TMD‐αvβ3 (~ 10%) and TMD‐GpA‐I expressers (~ 13%; Fig. 1B). Significant differences between all cell transfectants are given in Fig. 1B,C and the corresponding legends. These data indicated that the expression of a constitutively active αvβ3 endowed floating EOC cells with a higher cell viability and a delayed onset of apoptosis with an obvious advantage for cells grown suspended in ascites. In addition, Annexin‐Fluos V/PI staining was visualized by CLSM (see Fig. S1B). Next, we questioned whether the αvβ3 TMD conformational state alters the responsiveness of floating EOC cells to cisplatin. For this, the different cell transfectants were suspended for 30 h either in ascites or in DMEM (Fig. 1C), and then treated with cisplatin (2.5 μg·mL^−1^) for another 18 h. In TMD‐GpA and TMD‐αvß3 transfectants, cisplatin treatment led to a further appr. 5.6‐fold drop of their vital fractions. The vital fraction of the TMD‐GpA‐I transfectant dropped further by 2.9‐fold. The apoptotic fraction of TMD‐GpA cells under anoikis alone were further ~ 1.8‐fold enhanced by cisplatin, even ~ 65% of vital cells were already lost by anoikis alone. For TMD‐αvß3 cells, we noticed a ~ 3.5‐fold increased apoptotic fraction, considering that compared to TMD‐GpA, anoikis alone left 1.5‐fold more cells alive. For TMD‐GpA‐I transfectants, which retained upon anoikis alone 2‐ and 1.3‐fold more vital cells to be targeted by cisplatin than for TMD‐GpA and TMD‐αvß3 transfectants, respectively, its apoptotic fraction still remained lowest (~ 48%). For the necrotic cell fractions, we detected for TMD‐GpA‐I ~ 29%, for TMD‐αvβ3 expressers ~ 15%, and for TMD‐GpA ~ 20% (Fig. 1C). Cell culture in DMEM seemed to abrogate the differential chemoresponse among the cell transfectants, leading to a comparable drop in cell vitality ranging between ~ 29% and 35% and a comparable percentage of apoptotic cells ranging from ~ 51% to 60%, also in TMD‐GpA‐I expressing cells. Regarding the otherwise similar fraction size of necrotic TMD‐αvβ3 and TMD‐GpA‐I transfectants when compared to cell culture in the absence of cisplatin, only for TMD‐GpA expressers a drop of necrotic cell numbers by ~ 10% was noticed (Fig. 1C). Thus, in contrast to ascites as flotation medium, in DMEM, no significant differences between the different cell transfectants in each cell fraction were noticed.

### 3.2 Caspase activation as a function of the αvβ3 activation state

**Fig. 2:**
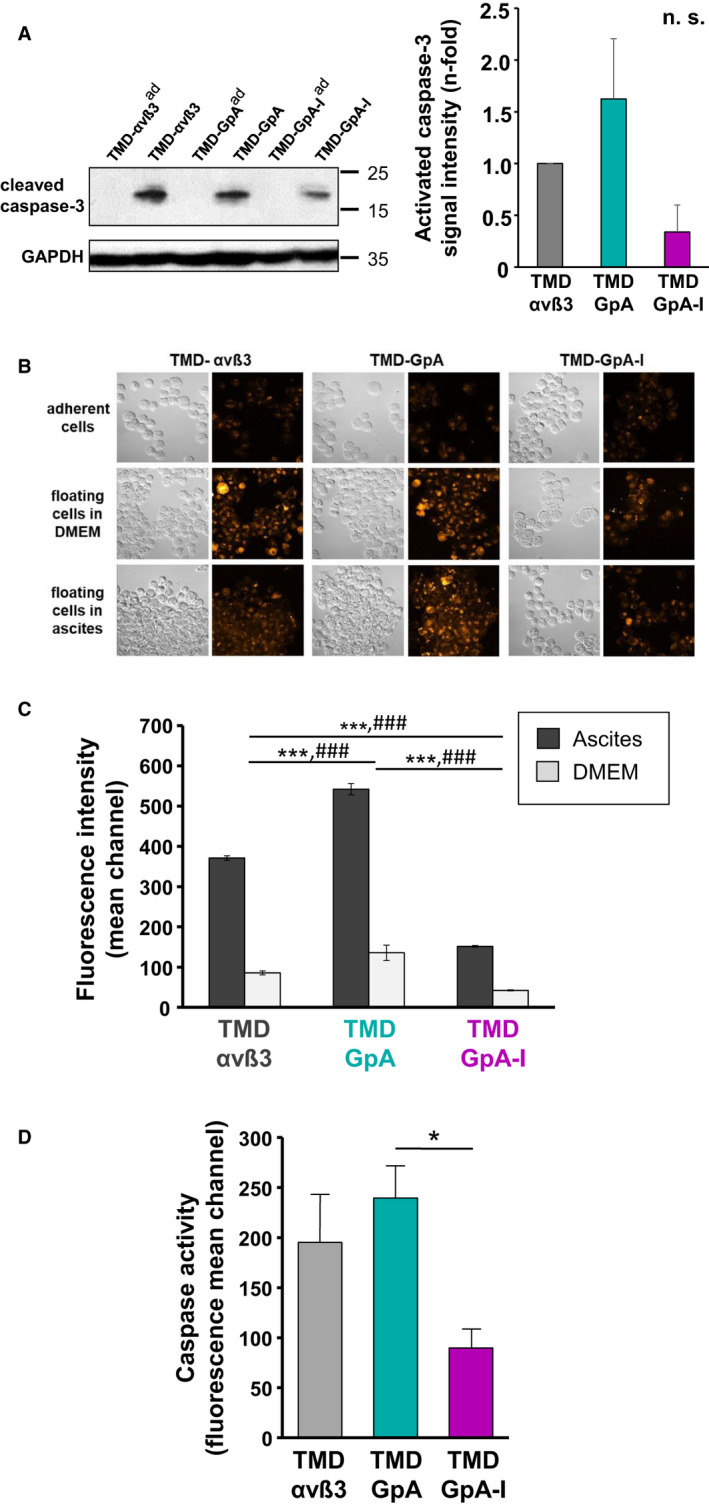
Caspase activation in human EOC cells expressing αvβ3 in different TMD conformational activation states. (A) Detection of activated caspase‐3 in floating vs. adherent αvβ3‐transfected EOC cells was done after 48 h of cultivation by western blot analysis. A representative western blot is depicted together with a histogram summarizing the mean values of GAPDH‐normalized fluorescence signal intensities ± SD (*n* = 3) as n‐fold, by setting the content of activated caspase‐3 in TMD‐αvβ3 expressers to ‘1’. Significance was tested by one‐way ANOVA on ranks with *post hoc* Tukey *t*‐test. Significance is indicated by *P < 0.050, **P < 0.010, and ***P < 0.001. Data did not reach significance (n. s.). (B) Immunocytochemical staining of activated caspase‐3 was done in adherent (ad) versus suspended cells and fluorescence signal intensities visualized by CLSM using an Alexa 488‐conjugated secondary Ab. Depicted are representative differential interference contrast images and the corresponding fluorescence images by using the look‐up table (LUT) ‘orange to white’ to indicate signal intensity according to the shown colored bar (scale bar: 50 µm). (C) FACS analysis of activated caspases was done by binding of a fluorescence‐labeled irreversible pan‐caspase inhibitor to cells suspended in ascites or DMEM for 48 h. Data are given as mean values of fluorescence mean channel ± SD (*n* = 3). Significance was tested by two‐way ANOVA with *post hoc* Bonferroni *t*‐test. The symbols * was used to indicate significant differences in ascites and ^#^ for medium. Significance is indicated by ^*/#^ *P* < 0.050, **^/##^ *P* < 0.010, and ***^/###^ *P* < 0.001. (D) Caspase activation was further determined indirectly by measuring its cleavage products of cytokeratin‐18 by FACS analysis. Data are given as mean values of fluorescence mean channel ± SD (*n* = 3). Significance of data was tested by one‐way ANOVA on ranks with *post hoc* Tukey *t*‐test. Significance is indicated by *P < 0.050, **P < 0.010, and ***P < 0.001.

Detection of cleaved and thus activated caspase‐3 by western blot analysis revealed in TMD‐GpA‐I transfectants a not significant ~ 3‐fold difference when compared to TMD‐αvβ3 and a ~ 4.6‐fold decreased caspase‐3 activation compared to TMD‐GpA expressers, which was close to significance (*P* = 0.065). In contrast, in all cell transfectants grown adherently, activated caspase‐3 levels were below detection limit (Fig. 2A). Immunocytochemical staining of cleaved caspase‐3 confirmed the results of the western blot analyses. In TMD‐αvβ3 and TMD‐GpA transfectants floating in DMEM, we observed comparable activated caspase‐3 levels, however, with markedly lower signal intensity in TMD‐GpA‐I expressers. TMD‐αvβ3 and TMD‐GpA‐I transfectants floating in ascites displayed similar levels, whereas highest signals were noticed for TMD‐GpA transfectants (Fig. 2B). Caspase activity was further measured by determining the binding of a fluorescence‐labeled irreversible pan‐caspase inhibitor. In principal, cell transfectants floating in DMEM showed lower inhibitor binding than those suspended in ascites with lowest levels in TMD‐GpA‐I transfectants, which were reduced by ~ 2.5‐ and ~ 3.6‐fold when compared to TMD‐αvβ3 and TMD‐GpA expressers, respectively. Cells floating in ascites exhibited higher levels of activated caspases, with a similar ~ 2.3‐ and ~ 3‐fold lower content in TMD‐GpA‐I when compared to TMD‐αvβ3 and TMD‐GpA expressers, respectively (Fig. 2C). Caspase‐3 activation was in addition determined indirectly by measuring its cleavage products of cytokeratin‐18. In TMD‐GpA‐I transfectants, we noticed a ~ 2.3‐ and ~ 2.8‐fold decrease in cytokeratin‐18 fragments when compared to TMD‐αvβ3 and TMD‐GpA transfectants, respectively (Fig. 2D). Significant differences between all cell transfectants are depicted in Fig. 2C,D.

### 3.3 Cell proliferation and spheroid formation by detached EOC cells expressing different αvβ3‐TMD variants

**Fig. 3:**
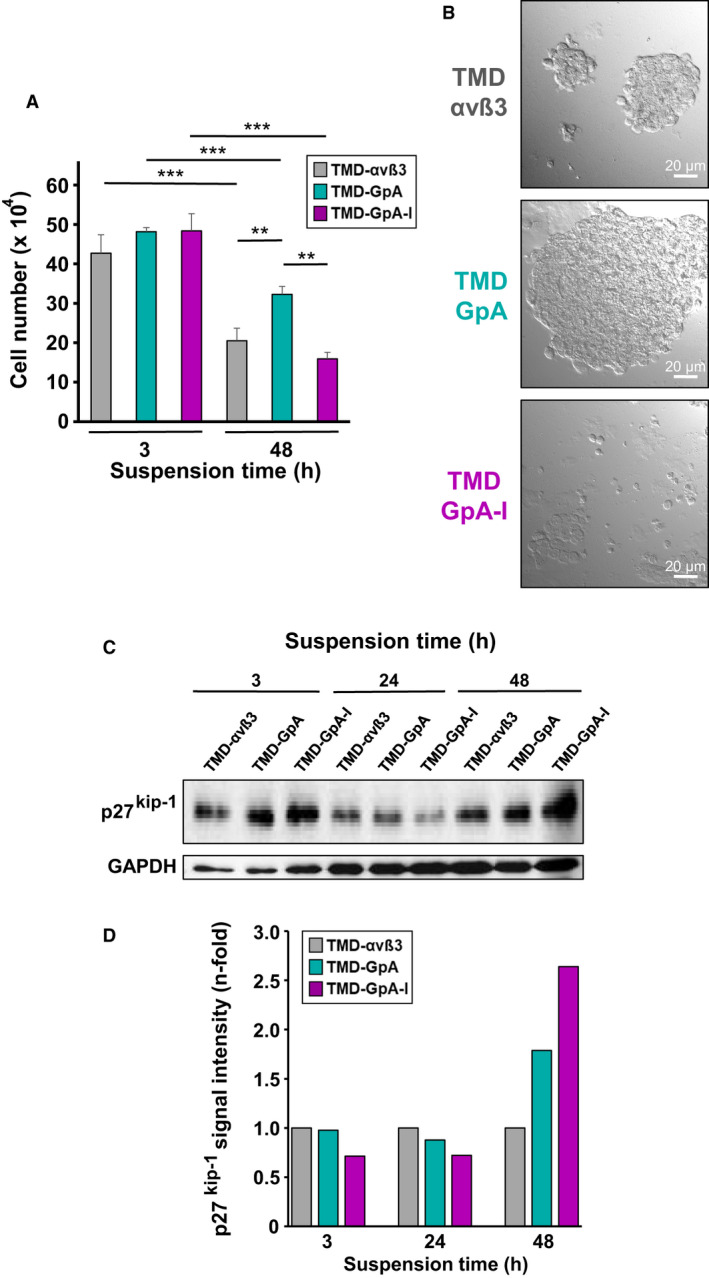
Proliferative activity of human EOC cells grown in suspension as a function of the αvβ3 activation state. (A) EOC αvβ3 cell transfectants floating in ascites were harvested after 3 and 48 h and cell numbers counted. Data are given as mean values of cell numbers ± SD (*n* = 3). Significance was tested by two‐way ANOVA with *post hoc* Bonferroni *t*‐test. Significance is indicated by *P < 0.050, **P < 0.010, and ***P < 0.001. (B) Formation of spheroid‐like cell clusters by TMD‐αvβ3, TMD‐GpA, and TMD‐GpA‐I, respectively, suspended for 48 h in ascites. Depicted are typical differential interference contrast images (scale bar: 20 µm). (C) Detection of p27^Kip1^ in αvβ3 cell transfectants suspended for 3, 24, and 48 h, respectively, in ascites. Shown is a representative western blot and the corresponding histogram depicting GAPDH‐normalized fluorescence signal intensities for p27^Kip1^ as n‐fold, by setting the values for TMD‐αvβ3 expressers at each time point to “1”.

Proliferative activity of suspended EOC cells in ascites was analyzed after 3 and 48 h. For all cell transfectants, we documented a marked decline of cell numbers after 48 h compared to that at 3 h. However, the extent of cell loss was highest in TMD‐GpA‐I transfectants (by ~ 67%), followed by that in TMD‐αvβ3 expressing cells (by ~ 50%), and by ~ 33% for TMD‐GpA transfectants (Fig. 3A).

In view of the tendency of EOC cells to form multicellular spheroid‐like aggregates upon flotation in ascites, after 48 h, we disclosed striking differences in cell cluster sizes among all cell transfectants. Whereas TMD‐GpA‐I expressers displayed only small cell clusters with still many single floating cells, TMD‐αvβ3 transfectants exhibited already larger cell aggregates, followed by tremendously increased cell cluster sizes formed by TMD‐GpA transfectants (Fig. 3B). In agreement with their lowest proliferative activity, TMD‐GpA‐I expressers exhibited highest expression levels of the cyclin/cyclin‐dependent kinase inhibitor p27^kip‐1^, which was increased by ~ 2.6‐ and ~ 1.7‐fold when compared to TMD‐αvβ3 and TMD‐GpA expressers, respectively (Fig. 3C).

**Fig. 4:**
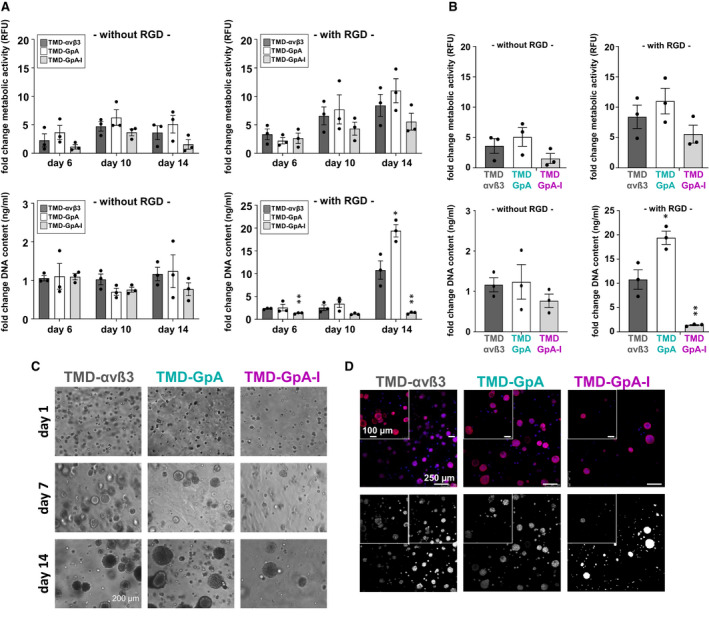
Proliferative activity of EOC cells grown in 3D‐hydrogels. (A) Cells were seeded in 3D‐hydrogels, which were polymerized in the absence or presence of RGD peptides, and maintained in 3D‐culture in 24‐well plates for 6, 10, and 14 days, respectively. Processing of samples for proliferation assays, AlamarBlue tests for the detection of cell metabolic activity, and CyQuant tests for DNA quantification were done as described [38]. Data are given as ‘fold change metabolic activity’ and ‘fold change DNA content’ from triplicate hydrogel samples in three biological replicates after normalization to day 1. Statistical analysis was done by independent *t*‐tests by considering *P*‐values ≤ 0.05 as statistically significant differences (*P < 0.05; **P < 0.01). (B) Proliferative activity of EOC cells grown in 3D‐hydrogels at day 14 (see A). (C) Morphology of the different cell transfectants grown in 3D‐hydrogels was assessed by bright‐field microscopy. Depicted are representative images after 1, 7, and 14 days of 3D‐culture, respectively, in hydrogels which were copolymerized with RGD peptides (scale bar: 200 µm). (D) Cell morphology was visualized by immunofluorescence as described under Materials and methods. Depicted are representative images after 14 days of 3D‐culture in RGD peptide‐containing hydrogels. Z‐stacks were acquired as described under Materials and methods. Depicted are representative images of cells grown for 14 days in 3D hydrogels (overview scale bar: 250 µm, zoom scale bar: 100 µm). Top row: illustrated in different colors: red: rhodamine 415‐conjugated phalloidin for the staining of F‐actin filaments; blue: DAPI for nuclei staining; bottom row: Color images were converted into black and white projections for better visualization.

In addition, EOC cell proliferation was assessed by seeding cells onto 3D‐hydrogels for 6, 10, and 14 days, respectively, and measuring the metabolic activity and the DNA content in the absence and presence of copolymerized RGD motifs [38]. In the presence of RGD motifs, at day 6, we did not observe any considerable difference in metabolic activity among all cell transfectants, which changed after another 4 days where TMD‐GpA‐I expressers already disclosed a drastic decline by ~ 34% and ~ 44% when compared to cells expressing TMD‐αvß3 and TMD‐GpA, respectively. After 14 days, lowest metabolic activity was still noticed for TMD‐GpA‐I expressers, whereas TMD‐αvß3 and TMD‐GpA transfectants continued to exceed cells expressing TMD‐GpA‐I by ~ 34% and ~ 49%, respectively. In the absence of RGD motifs, already at day 6, the TMD‐GpA‐I transfectant exhibited the lowest metabolic activity, which was reduced by ~ 50% and ~ 69% when compared to TMD‐αvß3 and TMD‐GpA transfectants, respectively. After 10 days of 3D‐culture, the metabolic activity increased further in all transfectants, keeping the relative differences among them very similar to that noticed at day 6. After 14 days, all transfectants disclosed a lower metabolic activity when compared to that on day 10. However, TMD‐GpA‐I transfectants showed an ~ 2.4‐fold decrease when compared to day 10 returning to an activity level already observed at day 6 (Fig. 4A,B). Using the DNA content as measure of cell proliferation, in the presence of RGD motifs, no significant differences were found among all transfectants at day 6. However, a significant increase was noticeable for TMD‐αvß3 and TMD‐GpA expressers by ~ 4.2‐fold and ~ 5.7‐fold, respectively, at day 14 over that at day 10 (and over day 6 by ~ 4.7‐ and ~ 7.6‐fold). In TMD‐GpA‐I transfectants, it remained at the level already detected at days 6 and 10, respectively. In the absence of RGD motifs, for all transfectants, a similar DNA content was measured which did not change over 14 days. Bright‐field imaging of the 3D‐cultures confirmed equal cell seeding densities for all transfectants at day 1 and spheroid formation over 14 days, with TMD‐GpA expressing cells resulting in the largest spheroid sizes consistent with the highest increase in cell proliferation in the presence of RGD motifs (Fig. 4C). CLSM images on day 14 also showed an increased capacity to form spheroid‐like cell clusters for both, TMD‐αvß3 and TMD‐GpA expressers, when compared to TMD‐GpA‐I expressing cells, which formed fewer spheroids in line with their lower proliferative activity (Fig. 4D).

### 3.4 EOC cell adhesion following a period of anchorage‐independent growth as a function of the αvβ3 activation state

**Fig. 5:**
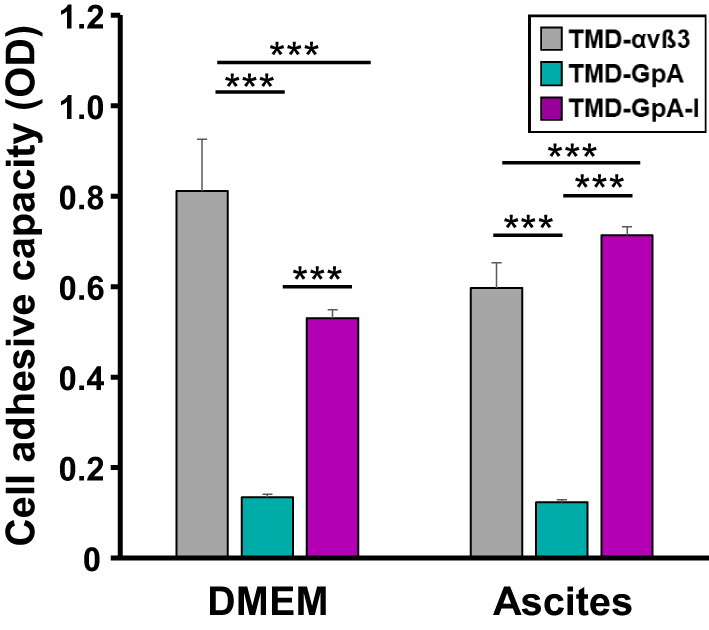
Cell adhesive capacity after anchorage‐independent cell growth in ascites or DMEM. The different αvβ3 cell transfectants were harvested from the suspensions after 48 h, seeded for 2 h onto cell culture plates, and their adhesive capacity determined as described. Data are given as mean values of optical density (OD) ± SD (*n* = 4). Significance was tested by two‐way ANOVA with *post hoc* Bonferroni *t*‐test. Significance is indicated by *P < 0.050, **P < 0.010, and ***P < 0.001.

During intraperitoneal metastasis, shed EOC cells floating in ascites have eventually to regain their adhesive capacity in order to establish secondary lesions. Thus, we asked whether the flotation period and the αvβ3 activation state influence this readhesion process. Cell transfectants were cultured for 48 h under nonadherent conditions in ascites or DMEM, withdrawn from the suspension, and allowed to adhere onto cell culture plates. Significant differences were noticed: TMD‐GpA transfectants displayed a low adhesive capacity in both culture media. In contrast, both, TMD‐αvβ3 and TMD‐GpA‐I transfectants, recovered efficient adhesive properties resulting in firm adhesion, which was highest in TMD‐αvβ3 expressers kept floating in DMEM, followed by TMD‐GpA‐I. Suspended in ascites, cells expressing TMD‐GpA‐I displayed the strongest adhesive capacity exceeding TMD‐GpA transfectants by ~ 6‐fold and TMD‐αvβ3 by ~ 1.2‐fold (Fig. 5).

### 3.5 EGF‐R expression and activation in detached EOC cells as a function of the αvβ3 activation state

**Fig. 6:**
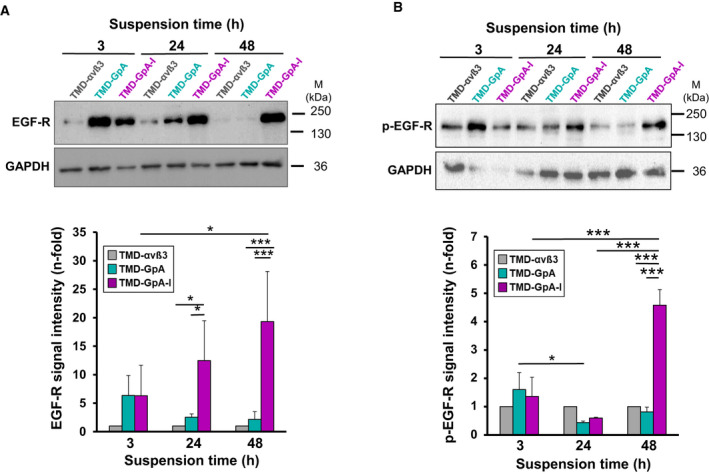
Expression and activation of the EGF‐R in EOC αvβ3‐TMD transfectants grown suspended in ascites. The expression of total EGF‐R (A) and activated EGF‐R (p‐EGF‐R) (B) was detected by western blot analysis after cell suspension for 3, 24, and 48 h, respectively. Depicted are typical western blots together with corresponding histograms each summarizing the mean values of GAPDH‐normalized fluorescence signal intensities ± SD (*n* = 3) by setting the values obtained for TMD‐αvβ3 transfectants at each time point to ‘1’. Significance was tested by two‐way ANOVA with *post hoc* Bonferroni *t*‐test. Significance is indicated by *P < 0.050, **P < 0.010, and ***P < 0.001.

For the detection of possibly altered EGF‐R expression and activation under anchorage‐independent growth, the different cell transfectants were grown for 3, 24, or 48 h suspended in ascites and (p‐) EGF‐R expression determined by western blot analysis. In TMD‐GpA‐I transfectants, we found a gradual increase in EGF‐R expression, reaching at 24 h a significant ~ 12‐fold and at 48 h a ~ 18‐fold higher level than TMD‐αvβ3 transfectants. On the opposite, in TMD‐GpA expressers, at 3 h of flotation, the EGF‐R content was almost comparable to that in TMD‐GpA‐I transfectants compared to markedly lower levels in TMD‐αvβ3 expressers. However, in TMD‐GpA expressers, the EGF‐R content significantly dropped over time still reaching a ~ 2‐fold higher level than TMD‐αvβ3 after 24 h with no further change until 48 h (Fig. 6A). Similar results were obtained for EGF‐R activation. In TMD‐GpA‐I transfectants—while showing a decline at 24 h—at 48 h, p‐EGF‐R levels were significantly up to ~ 4.7‐fold higher than in TMD‐αvβ3 and TMD‐GpA expressing cells, respectively (Fig. 6B).

### 3.6 Activation of integrin‐related signaling molecules in detached EOC cells expressing αvβ3 in its different activation states

**Fig. 7:**
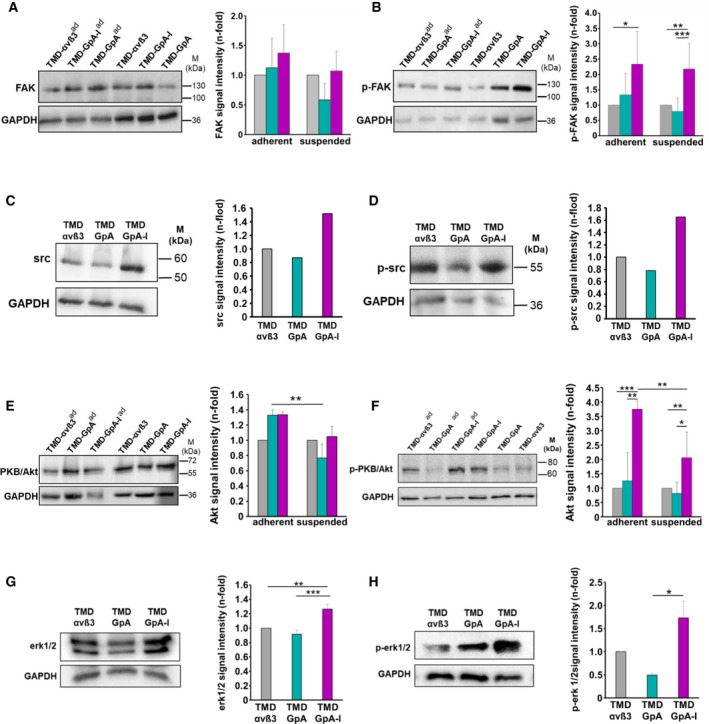
Expression and activation of integrin‐related signaling molecules in EOC αvβ3‐TMD transfectants. The expression levels of FAK (A), p‐FAK (B), PKB/Akt (E), and p‐PKB/Akt (F), respectively, were detected in cells grown suspended or adherent (ad) to cell culture plates for 48 h, by western blot analysis. Typical western blots are depicted together with corresponding histograms summarizing the mean values of GAPDH‐normalized fluorescence signal intensities ± SD from a minimum of 3 independent experiments as n‐fold, by setting the values obtained for TMD‐αvβ3 expressers under each culture condition to ‘1’. For the expression of src (C) and p‐src (D) representative western blots are shown together with histograms outlining the corresponding relative signal intensities by setting that in TMD‐αvβ3 to ‘1’. The kinases p44/p42^erk1/2^ (G), and p‐p44/p42^erk1/2^ (H), respectively, were determined by western blot analysis in cell transfectants cultured for 48 h suspended in ascites as described under (A). Representative western blots are depicted together with the corresponding histograms from 3 independent experiments as described under (A). Significance was tested by two‐way ANOVA with *post hoc* Bonferroni *t*‐test. Significance is indicated by *P < 0.050, **P < 0.010, and ***P < 0.001.

In order to study integrin‐triggered cell signaling as a function of the αvβ3 TMD conformational state, we first measured the expression of the downstream kinase FAK [21]. Except a slight but not significant FAK decrease in suspended TMD‐GpA expressers, TMD‐GpA‐I and TMD‐αvβ3 transfectants revealed very similar levels, also in adherently grown cells (Fig. 7A). However, for activation of FAK, a significant up to ~ 2.5‐fold increase was exclusively documented in TMD‐GpA‐I transfectants over TMD‐αvβ3 and TMD‐αvβ3 transfectants. In adherent TMD‐GpA‐I transfectants, we confirmed the previously shown FAK activation [32] (Fig. 7B). The expression and activation of the nonreceptor tyrosine kinase Src, which also affects integrin‐mediated signaling and is linked to enhanced tumor cell growth, metastasis, and survival [22, 23, 39, 40, 41], was up to ~ 2‐fold increased in TMD‐GpA‐I expressers over TMD‐αvβ3 and TMD‐GpA transfectants, after a flotation period of 48 h in ascites (Fig. 7C,D). The expression of the serine‐threonine kinase PKB/Akt, which is activated by FAK following integrin‐mediated cell adhesion and plays a critical role in integrin signaling [24], was not prominently changed among all transfectants. However, in TMD‐GpA‐I expressers, it was activated by up to ~ 2.3‐ and ~ 2.7‐fold compared to TMD‐αvβ3 and TMD‐GpA transfectants, respectively (Fig. 7E,F). Moreover, in TMD‐GpA‐I transfectants, we determined an only slight but significant increase in p44/p42^erk1/2^ over that in TMD‐αvβ3 and TMD‐GpA expressers, respectively (Fig. 7G). Activation of p44/p42^erk1/2^ was significantly elevated in TMD‐GpA‐I over TMD‐GpA transfectants (by up to ~ 3‐fold), with a slight difference to TMD‐αvβ3 (~1.7‐fold; Fig. 7H).

### 3.7 Expression of Bcl‐2 and survivin in detached EOC cells as a function of the αvβ3 activation state

**Fig. 8:**
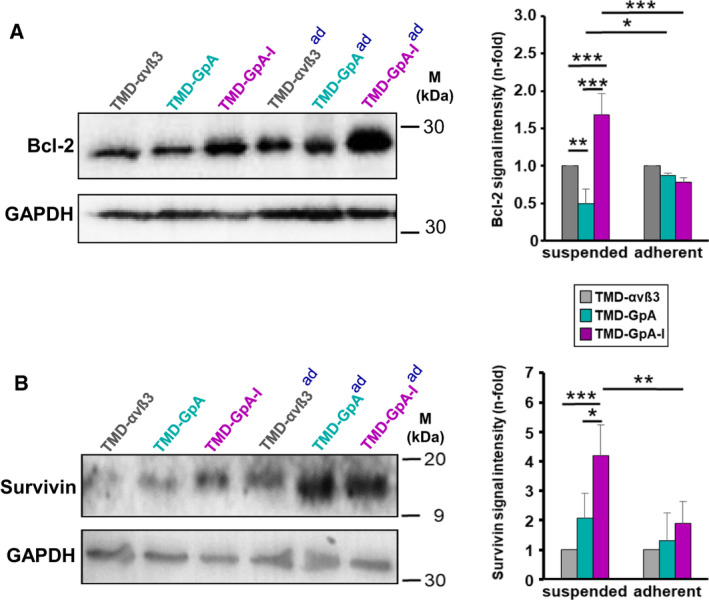
Expression of Bcl‐2 and survivin in EOC cells depending on the αvβ3‐TMD conformational activation states. Bcl‐2 (A) and survivin (B) expression was detected by western blot analysis in cells cultured in suspension or under adherence (ad) for 48 h. Representative western blots are depicted together with histograms illustrating the mean values of GAPDH‐normalized fluorescence signal intensities ± SD (*n* = 3) as n‐fold, by setting the values obtained for TMD‐αvβ3 transfectants under each culture condition to ‘1’. Significance was tested by two‐way ANOVA with *post hoc* Bonferroni *t*‐test. Significance is indicated by *P < 0.050, **P < 0.010, and ***P < 0.001.

Based on the observed prolonged survival and delayed onset of apoptosis of detached EOC cells expressing TMD‐GpA‐I, we explored next, whether a differential effect occurred on the expression of the anti‐apoptotic proteins Bcl‐2 [14] and survivin [42]. Bcl‐2 and survivin expression was determined by western blot analysis after 48 h of cell culture either suspended in ascites or adherently grown. In detached TMD‐GpA‐I expressers, Bcl‐2 expression showed a significant ~ 2‐ and ~ 4‐fold increase over levels in TMD‐αvβ3 and TMD‐GpA transfectants, respectively (Fig. 8A). In adherent cells, we did not notice any difference in Bcl‐2 levels. The levels of the anti‐apoptotic protein survivin in floating TMD‐GpA‐I transfectants significantly exceeded those in TMD‐αvβ3 and TMD‐GpA by ~ 4‐ and ~ 2.7‐fold, respectively. Moreover, compared to adherent TMD‐GpA‐I expressers, survivin expression was ~ 2‐fold enhanced when cells were kept under suspension. The survivin content among all adherent cell transfectants did not display significant differences (Fig. 8B).

## 4 Discussion

### 4.1 Influence of the αvβ3 activation state on spheroid formation, anoikis escape, and chemoresistance of anchorage‐independent EOC cells

We explored EOC cell survival upon loss of anchorage as a function of the αvβ3 TMD conformational activation state by the use of the above described well‐characterized EOC cell model [32]. Suspension of TMD‐GpA‐I expressers in ascites conferred prolonged cell survival and delayed onset of anoikis, whereas TMD‐GpA expression provoked an obvious drop in cell viability, concomitant with a higher amount of apoptotic and necrotic cells. In line with this, TMD‐GpA‐I expression markedly decreased caspase‐3 activation compared to TMD‐αvβ3 and especially TMD‐GpA transfectants. In accordance, we found lowest binding activity of a pan‐caspase inhibitor in TMD‐GpA‐I. This observation opposes the notion of the occurrence of the IMD in *per se* anchorage‐dependent epithelial‐type cells, in case of unligated integrins [43]. However, in transformed malignant cells, various oncogenes and yet not fully resolved events may provoke constitutive integrin activation, including somatic mutations in integrin TMD and cytoplasmic regions. This is thought to affect function switches of unligated integrins, contributing to anchorage‐independence, prosurvival signaling, and anoikis resistance [5, 44, 45] in line with the prolonged anchorage‐independent cell survival observed by us for the constitutively active αvβ3 variant TMD‐GpA‐I. In fact, there is evidence for constitutively activated integrins from other cancer cells during tumor progression. In breast cancer cells, full αvβ3 activation was instrumental for their hematogenous spread and homing in bone [46, 47]. In prostate cancer cells lacking β3, the reintroduction of a β3 mutant encompassing a point mutation (D723R) disrupting a putative salt bridge in the α‐ and the β‐cytoplasmic domains locked αvβ3 in an activated state capable of triggering constitutive FAK activation [48]. The release of this cytoplasmic constraint in the platelet integrin αIIbβ3 also conferred constitutive cell signaling properties [49]. In breast cancer cells, αvβ3 exists in a nonactivated, a less activated, and, even in the absence of an exogenous ECM ligand, an increased activated state [50]. In ascites‐derived mouse leukemia cells, highly and constitutively activated conformations for β1, β2, and β3 integrins had been detected, also suggesting a dysregulated *inside‐out* signaling [51].

The acquisition of chemoresistance, going along with changes in integrin‐mediated cell adhesion and cytoskeletal organization, represents a major treatment challenge [52, 53, 54]. Expectedly, we found that cisplatin caused a marked drop of cell viability in all cell transfectants, but still, TMD‐GpA‐I transfectants harbored the largest fraction of viable cells and a smaller percentage of apoptotic cells, suggesting a dominant impact of a constitutively active αvβ3 on resistance to this drug. A role of αvβ3 in cell survival, apoptosis, and chemoresistance had been reported before in glioma [55], breast cancer cells against taxol‐induced apoptosis [56] or epirubicin [58], and in laryngeal cancer cells against cisplatin [57], strengthening the notion of αvβ3 and even more, its constitutively active form, as a promising target against drug resistance [59].

By forcing these cell transfectants into suspension, they aggregated to multicellular clusters similar to EOC spheroids in ascites. The observation that TMD‐GpA‐I transfectants suspended in ascites displayed smallest cell clusters came unexpected, since we anticipated a survival advantage of spheroid‐like cell aggregates over single cells by enhanced cell–cell contacts to compensate for their loss of ECM anchorage. Possibly, the presence of a constitutively active integrin with full signaling competence overrules the need for strengthened cell–cell adhesion. However, for cells carrying TMD‐GpA, the formation of large cell aggregates appeared to be indispensable to fight anoikis. Next, we were interested, whether cells are capable of regaining adhesive properties following a cell flotation period in ascites. TMD‐GpA transfectants displayed lowest adhesive capacity in contrast to cells expressing either TMD‐αvβ3 or TMD‐GpA‐I which both re‐established firm cell adhesion as an important prerequisite for eventual reanchoring to an appropriate mesothelial ECM allowing metastatic colonization [8, 9, 11, 16].

### 4.2 Proliferative activity of anchorage‐independent EOC cells harboring αvβ3 in differently active TMD conformations

Mechanisms governing anoikis are intimately associated with progression of tumor cells through the cell cycle machinery. As such, many factors affecting cell proliferation also take over important functions in the onset of apoptosis by sensing if microenvironmental conditions permit proper cell division [60, 61]. Thus, G1 growth arrest is usually initiated, for example, when anchorage‐dependent cells are hindered to attach to an appropriate ECM [62]. Here, we disclosed lowest cell division capacity in TMD‐GpA‐I transfectants, followed by TMD‐αvβ3 expressing cells. In TMD‐GpA transfectants, we found highest cell numbers after 48 h of suspension in ascites. Based on the data from the apoptosis assays revealing the highest percentage of viable cells for TMD‐GpA‐I expressers when compared to that of TMD‐GpA, one may assume that proliferation slowed down as a function of TMD‐GpA‐I, leaving—at least in part—cells in a quiescent state. Reduced proliferative activity of TMD‐GpA‐I transfectants was confirmed in a 3D‐hydrogel culture model [38]. In the presence of RGD motifs, after 14 days, we noticed lowest metabolic activity for TMD‐GpA‐I expressers. In the absence of RGD motifs, TMD‐GpA‐I transfectants exhibited already after 6 days lowest metabolic activity. After 14 days, the metabolic activity of all transfectants was still low, with TMD‐GpA‐I transfectants expressing similar levels like at the beginning of the observation period. The DNA content in the presence of RGD motifs was not significantly altered among all cell transfectants until day 10. After 14 days, it significantly increased in TMD‐αvß3 and TMD‐GpA expressers compared to day 10. TMD‐GpA‐I expressers, however, remained at the same level detected at days 6 and 10, respectively. In the absence of RGD motifs, a similar DNA content was measured for all cell transfectants which did not significantly change over 14 days. Like observed for floating EOC cell clusters in ascites, bright‐field and CLSM imaging of cells in 3D‐hydrogels showed smallest spheroid sizes over 14 days for TMD‐GpA‐I expressers, in line with their low proliferative activity. This was not unexpected, since the restraints put on EOC cells under anchorage‐independence may bring forth (sub‐) populations of cells, which exert low proliferative activity, at least in distinct layers of spheroid‐like structures. Hereby, the onset of apoptosis might be circumvented in case of inappropriate growth conditions, favoring cell survival during early stages of the metastatic spread, and consequently, also a weaker response to anti‐mitotic drugs [1, 63, 64]. Even spheroid shrinking is frequently observed due to partial drug‐induced tumor cell killing, their complete eradication is mostly not achieved [65, 66]. Most interestingly, p27^kip‐1^ expression, whose downregulation is involved in EOC progression [67], was elevated in the low proliferating TMD‐GpA‐I expressers. Also, in MCF‐10A mammary epithelial cells, anoikis resistance was found to be linked to cell cycle arrest by elevated p27^kip1^ expression [25]. Moreover, β1 integrins conferred anoikis resistance by reduced expression of p21 and p27^kip1^ [68], underlining the importance of the connection between cell cycle arrest and anoikis evasion.

### 4.3 Activation of the EGF‐R and integrin‐related signaling molecules as a function of the activation state of αvβ3 in floating EOC cells

Signals from integrins and the EGF‐R are intimately connected. Integrin engagement results in ligand‐independent EGF‐R activation and, *vice versa*, upon EGF‐R activation, integrins may signal independently from ligand engagement [69, 70, 71]. Also, they mutually control their expression levels suggesting a feed‐forward mechanism to enhance synergistic protumorigenic signaling [72, 73]. Indeed, in TMD‐GpA‐I transfectants, EGF‐R expression and activation significantly increased over time of observation, whereas TMD‐GpA expression led to a drastic drop. Upon loss of integrin engagement, normal cells decrease EGF‐R expression, resulting in suppressed prosurvival signaling, suggesting deregulated EGF‐R as a crucial player in anoikis escape mechanisms [74, 75]. Hereby, EOC cells may uncouple survival from adhesion via synergistic receptor crosstalk and signaling, provoking FAK stimulation and consequently, MAPK and PKB/Akt signaling [75, 76, 77]. Adherent normal epithelial cells sustain proliferation and viability by activating survival signaling pathways; however, during oncogenic cell transformation, constitutive activation of these pathways may be triggered [24, 26]. Exclusively in floating TMD‐GpA‐I transfectants, we found increased activation of FAK which is known to correlate with cancer aggressiveness caused by its role in anoikis escape [78, 79]. Continuous src activation was also shown to trigger constitutive FAK activation, allowing PI3K recruitment and subsequent PKB/Akt activation. This in turn suppressed anoikis by promoting the phosphorylation of the proapoptotic factor Bad and the blockade of caspases [80, 81]. In line with this, exclusively in TMD‐GpA‐I transfectants floating in ascites, src expression and activation were elevated, in agreement with previous findings in IMD‐resistant tumor cells, where unligated αvβ3 promoted anoikis resistance by src activation and recruitment to the cytoplasmic ß3 domain. In contrast to our findings, FAK seemed here not to be involved [22, 40]. In a positive feedback loop, src activates the EGF‐R even in the absence of an appropriate ligand, leading to the activation of the p44/p42^erk1/2^ and PKB/Akt pathways [22, 82, 83, 84, 85], aligning well with our observations on the activation of these signaling routes via EGF‐R/αvβ3 crosstalk [72]. Indeed, in suspended TMD‐GpA‐I transfectants, PKB/Akt activation was slightly but significantly increased. The role of PKB/Akt in anoikis escape has also been shown in epithelial cells via stimulated Bcl‐2 transcription [86, 87, 88]. In floating TMD‐GpA‐I transfectants, we also noticed a significant increase in activated p44/p42^erk1/2^ over TMD‐GpA expressers which is implicated in apoptosis resistance depending at least in part on αv‐integrins and elevated EGF‐R [89]. This suggests that EOC spheroids might exploit the activation of both pathways—in concert with elevated EGF‐R activity—to counteract loss of ECM/cell anchorage.

### 4.4 Impact of constitutive αvβ3 activation in anchorage‐independent EOC cells on the anti‐apoptotic factors Bcl‐2 and survivin

Upon loss of ECM attachment, Bcl‐2 levels were elevated in TMD‐GpA‐I compared to TMD‐αvβ3 and, even more, TMD‐GpA transfectants. This points to a contribution of αvβ3 signaling to anoikis avoidance via Bcl‐2 upregulation. In agreement with our data from floating TMD‐GpA‐I transfectants, the control over Bcl‐*2* expression in adherent cells was shown to depend on Src kinases and required the activation of ras by FAK, which in turn led to activated PI3K/Akt signaling. Also relating to our data, Bcl‐2 was shown to contribute to the control of cell cycle progression via the blockade of the transition between G0/G1 and S phases, at least in part, by modulating p27^kip1^ levels [90, 91]. Moreover, we found that survivin expression in TMD‐GpA‐I transfectants significantly exceeded that in TMD‐αvβ3 and TMD‐GpA expressers by several fold. In fact, survivin is known to be overexpressed in cancer upon activated FAK and PKB/Akt signaling correlating with poor patient prognosis due to chemoresistance and thus disease recurrence [54, 92, 93].

## 5 Conclusions

In conclusion, the use of our EOC αvβ3 transfected cell model allowed the investigation and direct comparison of differential αvβ3 effects depending on its distinct TMD conformational activation states. Our results underline the capability of a high‐affinity αvβ3 receptor displaying constitutive signaling competence to promote the activation of important players involved in anoikis resistance, including the EGF‐R, FAK, src, PKB/Akt, and MAPK, as well as the induction of anti‐apoptotic factors. To unravel the properties of unengaged constitutively active integrins during metastasis is of high clinical interest in order to further the understanding of the reasons for the frequent failures of integrin‐targeted clinical cancer trials.

## Conflict of interest

The authors declare that there are no conflicts of interest regarding the publication of this article.

## Author contributions

UR conceptualized and designed the study, methodology, validation, investigation, provided guidance and supervision for the investigators, and wrote the manuscript (original draft). RD and JH performed the experiments and analyzed the data. AB and ES were responsible for methodology of experimentation. DL performed and contributed the 3D‐cell experiments and reviewed/edited the manuscript. EKE reviewed and edited the manuscript. CF performed statistical data analyses.

## Supporting information


**Fig. S1.** Annexin‐Fluos V/PI FACS apoptosis assays.
**Fig. S2.** Western Blot analysis of caspase‐3, (p‐)EGF‐R, the integrin related signaling molecules (p‐)FAK, (p‐)src, (p‐)PKB/Akt, and (p‐)p44/42/erk1/2, as well as of the anti‐apoptotic factors Bcl‐2 and survivin.
