# Supplementary material for: Constitutive activation of integrin αvβ3 contributes to anoikis resistance of ovarian cancer cells
Source: Mol Oncol. 2020 Dec 1;15(2):503–22. doi: 10.1002/1878-0261.12845 (PMC7858284; doi:10.1002/1878-0261.12845)
Supplement: Supplementary file 1 — Fig. S1. Annexin‐Fluos V/PI FACS apoptosis assays. Fig. S2. Western Blot analysis of caspase‐3, (p‐)EGF‐R, the integrin related signaling molecules (p‐)FAK, (p‐)src, (p‐)PKB/Akt, and (p‐)p44/42/erk1/2, as well as of the anti‐apoptotic factors Bcl‐2 and survivin. [file MOL2-15-503-s001.pptx]

## Slide 1
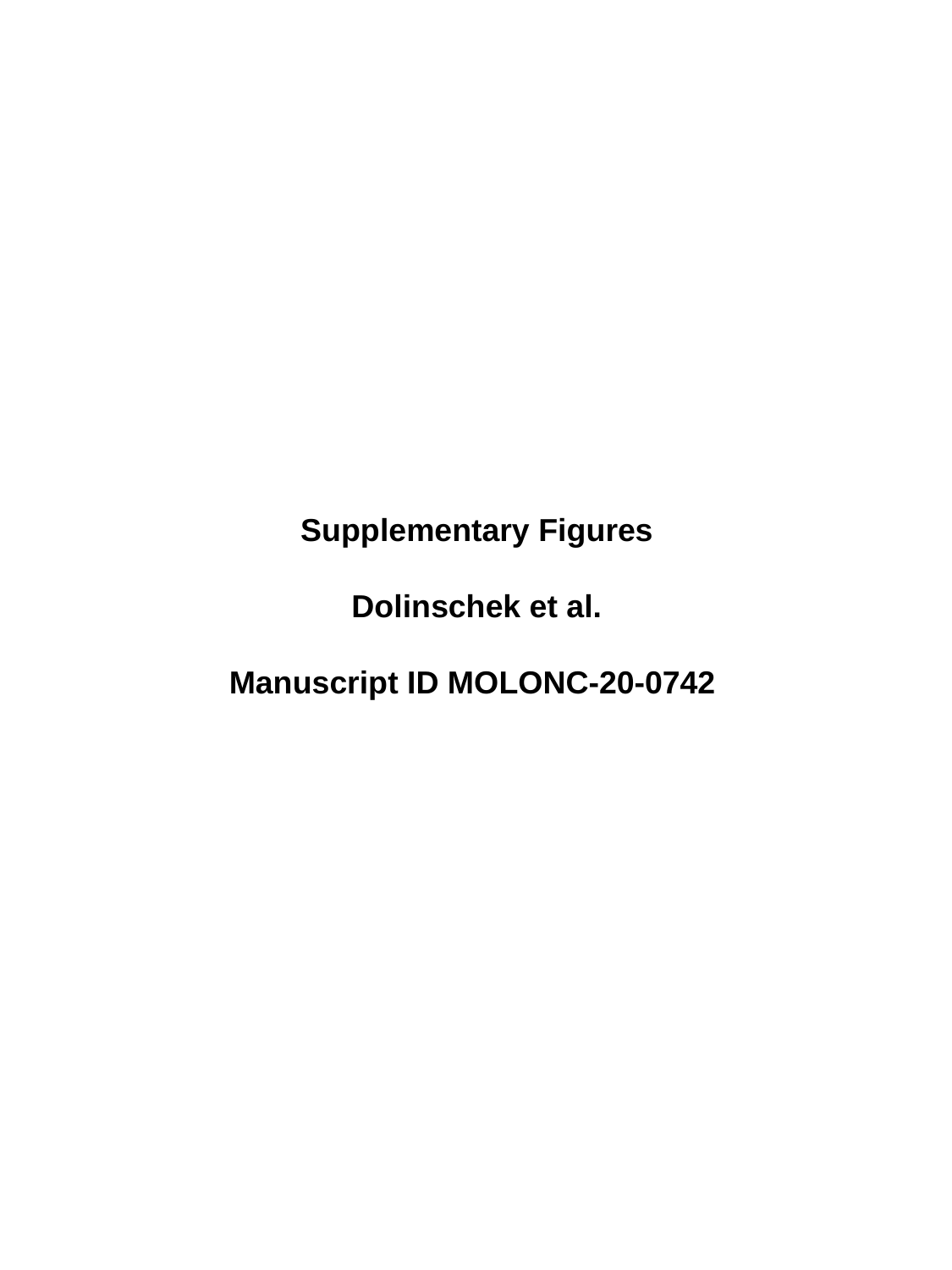

Supplementary Figures
Dolinschek et al.
Manuscript ID MOLONC-20-0742

## Slide 2
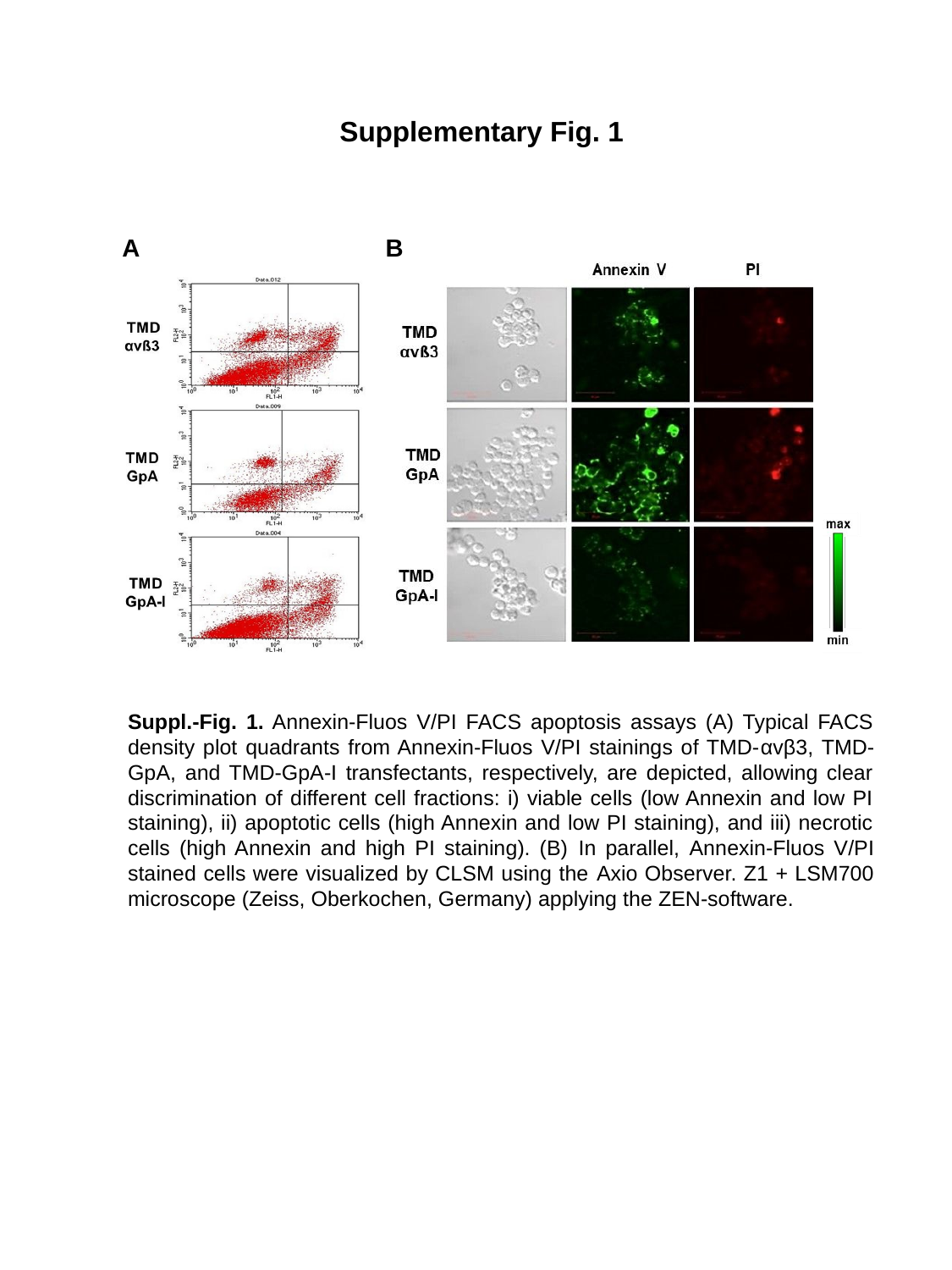

Supplementary Fig. 1
A
B
Suppl.-Fig. 1. Annexin-Fluos V/PI FACS apoptosis assays (A) Typical FACS density plot quadrants from Annexin-Fluos V/PI stainings of TMD-αvβ3, TMD-GpA, and TMD-GpA-I transfectants, respectively, are depicted, allowing clear discrimination of different cell fractions: i) viable cells (low Annexin and low PI staining), ii) apoptotic cells (high Annexin and low PI staining), and iii) necrotic cells (high Annexin and high PI staining). (B) In parallel, Annexin-Fluos V/PI stained cells were visualized by CLSM using the Axio Observer. Z1 + LSM700 microscope (Zeiss, Oberkochen, Germany) applying the ZEN-software.

## Slide 3
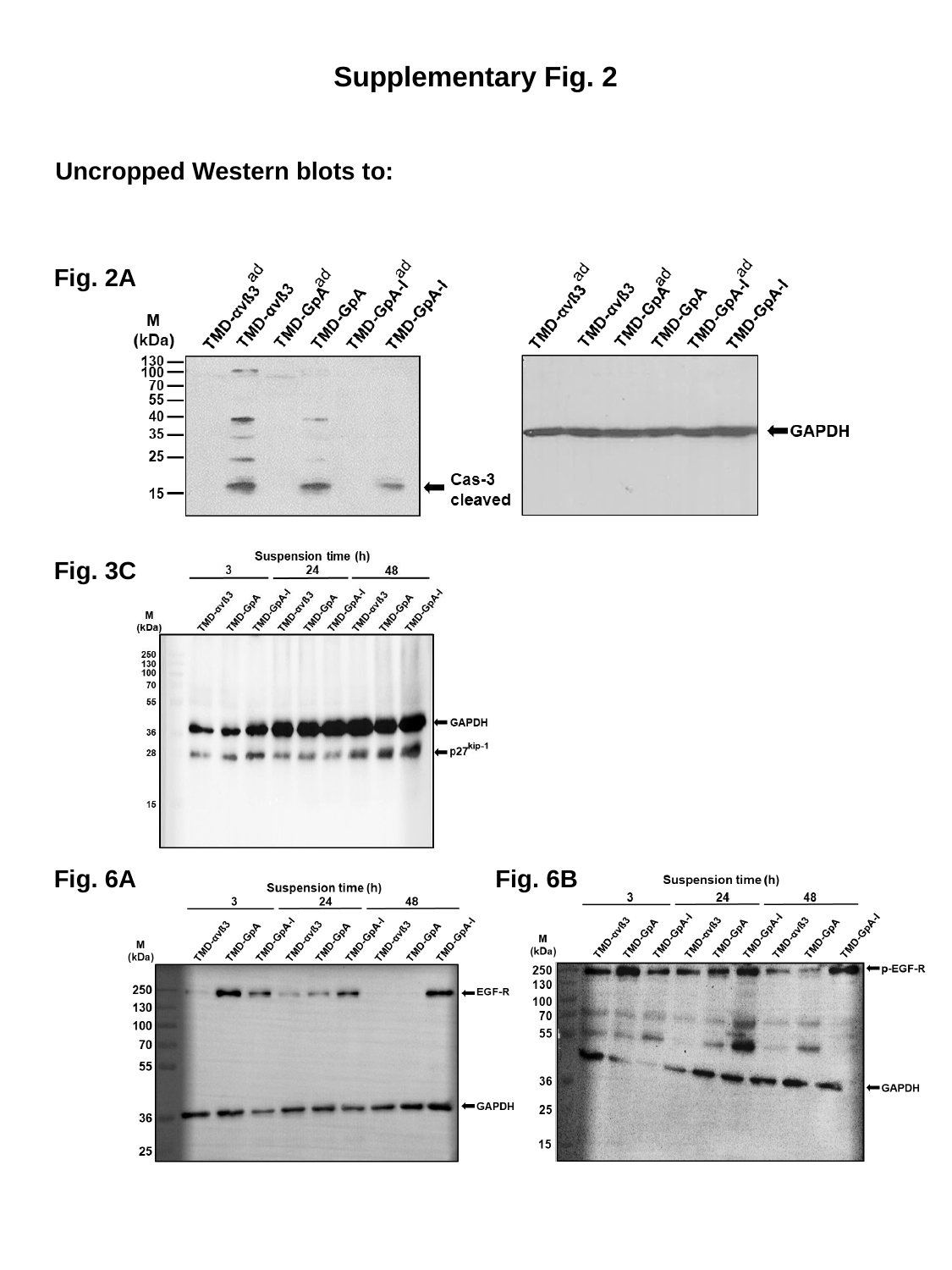

Supplementary Fig. 2
Uncropped Western blots to:
Fig. 2A
Fig. 3C
Fig. 6A
Fig. 6B

## Slide 4
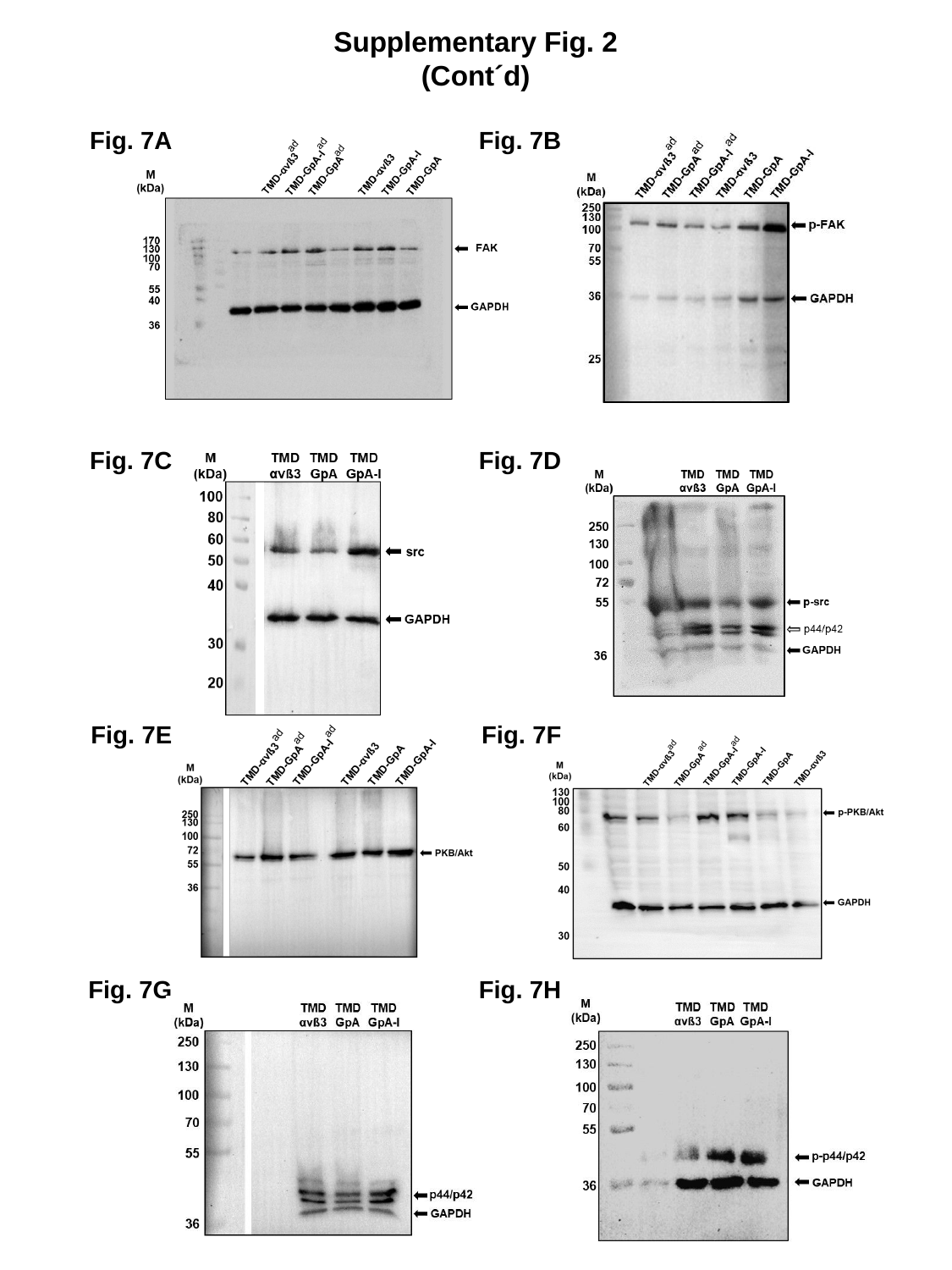

Supplementary Fig. 2
(Cont´d)
Fig. 7A
Fig. 7B
Fig. 7C
Fig. 7D
Fig. 7E
Fig. 7F
Fig. 7G
Fig. 7H

## Slide 5
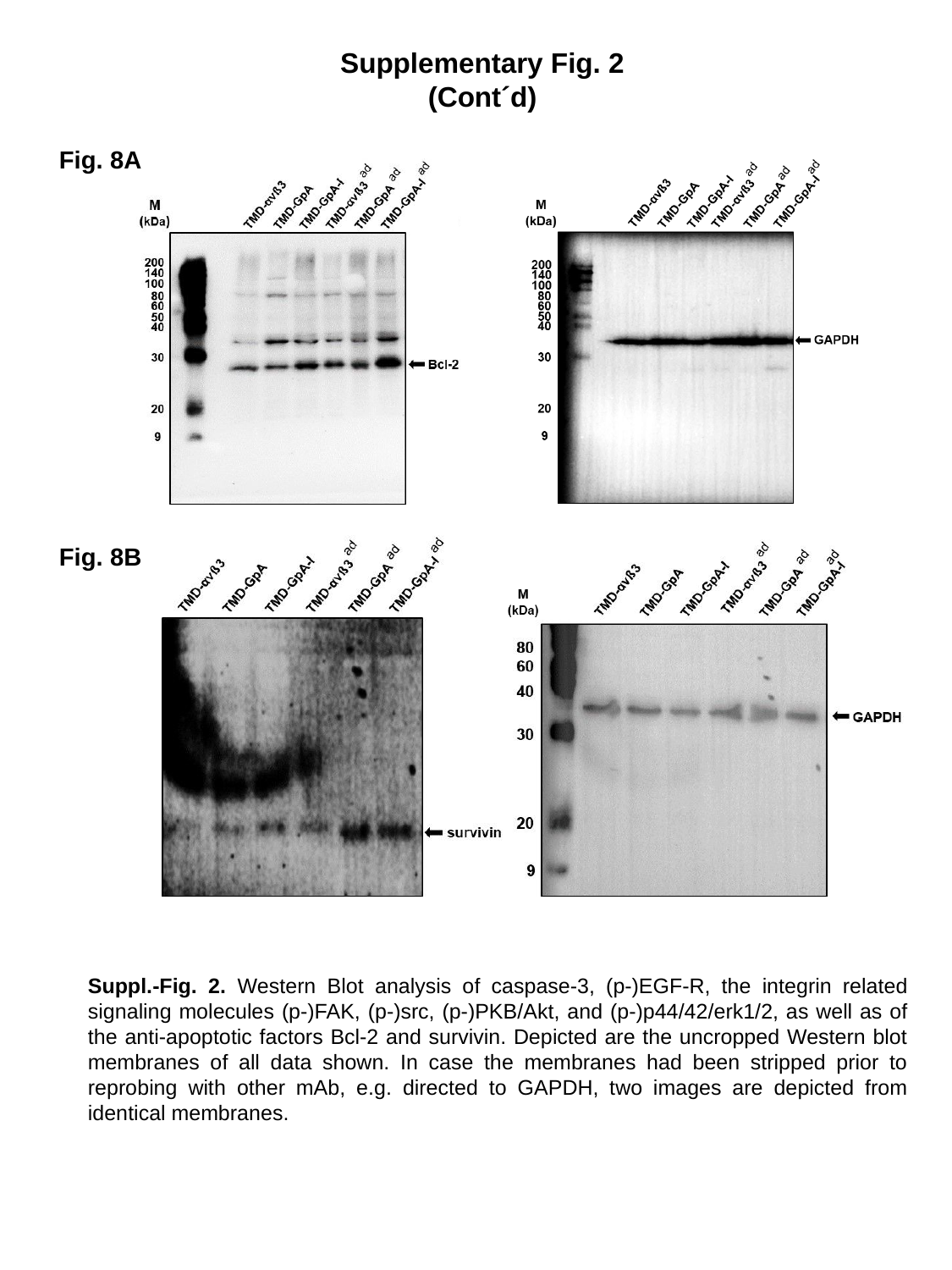

Supplementary Fig. 2
(Cont´d)
Fig. 8A
Fig. 8B
Suppl.-Fig. 2. Western Blot analysis of caspase-3, (p-)EGF-R, the integrin related signaling molecules (p-)FAK, (p-)src, (p-)PKB/Akt, and (p-)p44/42/erk1/2, as well as of the anti-apoptotic factors Bcl-2 and survivin. Depicted are the uncropped Western blot membranes of all data shown. In case the membranes had been stripped prior to reprobing with other mAb, e.g. directed to GAPDH, two images are depicted from identical membranes.
